# False Data Injection Impact on High RES Power Systems with Centralized Voltage Regulation Architecture

**DOI:** 10.3390/s23052557

**Published:** 2023-02-25

**Authors:** Tommaso Bragatto, Marco Antonio Bucarelli, Maria Sofia Bucarelli, Federico Carere, Alberto Geri, Marco Maccioni

**Affiliations:** 1 Department of Astronautic Electrical and Energy Engineering, Sapienza University of Rome, 00185 Rome, Italy; 2 Department of Computer, Control and Management Engineering, Sapienza University of Rome, 00185 Rome, Italy

**Keywords:** cybersecurity, false data, distributed generation, voltage regulation

## Abstract

The increasing penetration of distributed generation (DG) across power distribution networks (DNs) is forcing distribution system operators (DSOs) to improve the voltage regulation capabilities of the system. The increase in power flows due to the installation of renewable plants in unexpected zones of the distribution grid can affect the voltage profile, even causing interruptions at the secondary substations (SSs) with the voltage limit violation. At the same time, widespread cyberattacks across critical infrastructure raise new challenges in security and reliability for DSOs. This paper analyzes the impact of false data injection related to residential and non-residential customers on a centralized voltage regulation system, in which the DG is required to adapt the reactive power exchange with the grid according to the voltage profile. The centralized system estimates the distribution grid state according to the field data and provides the DG plants with a reactive power request to avoid voltage violations. A preliminary false data analysis in the context of the energy sector is carried out to build up a false data generator algorithm. Afterward, a configurable false data generator is developed and exploited. The false data injection is tested in the IEEE 118-bus system with an increasing DG penetration. The false data injection impact analysis highlights the need to increase the security framework of DSOs to avoid facing a relevant number of electricity interruptions.

## 1. Introduction

Nowadays, cybersecurity plays a major role in electric grid management. Traditionally, electrical systems were controlled using serial devices connected to computers with proprietary protocols; meanwhile, today’s control systems are increasingly connected to more extensive enterprise networks, which can expose these systems to similar vulnerabilities that are typically found in computer systems. Therefore, cybersecurity has become a crucial challenge for realizing safe and reliable smart grids [1,2,3,4]. The main cybersecurity threat vectors, through which the malicious attacker can gain access to a device or a control network, are external users accessing the network through the Internet, misconfigured firewalls, unsecured wireless routers and wired modems, infected laptops located elsewhere that can access the network behind the firewall, infected USB keys and PLC logic programs, and unsecured RS-232 serial links [5]. This paper assesses the effects of a VR regulation mechanism when false data are measured. Therefore, the surveyed literature firstly surveys the references about voltage regulation; secondly, other works related to the effects of false data are collected. 

The worldwide increase in cybersecurity attacks in the energy sector has been accompanied by the deep penetration of distribution generator (DG) systems among electrical grids, referring to photovoltaic (PV) and wind power plants. Increasing the exploitation of communication networks to manage a more complex system has generated new vulnerabilities to malicious threats in the system [6,7,8,9]. More information must be transmitted and processed to improve interoperability and efficiency for predicting and controlling power generation, consumption, and storage [10]. The transition from a passive grid to an active grid, characterized by high renewable energy source (RES) penetration, involves facing technical challenges, such as the presence of relevant RPF [11], voltage stability [12], an increase in the number of interruptions [13], etc.

In this context, investigations on false data injection attacks (FDIAs) on smart grids have gained relevance. Reference [14] highlights that most of the attacks on smart grids usually lead to false data injection; then, many detection algorithms are developed, as in [15,16,17,18], while evaluating the effects of false data on power systems is a less investigated topic. Moreover, FDIA detection algorithms are often tested against manually calculated anomalous profiles that do not consider probabilistic scenarios, as in [17]. Therefore, this study aims to investigate how randomly generated false data could impact a smart distribution network that adopts a centralized voltage regulation (VR) framework to mitigate the effects of distributed generation. 

In the literature, many papers have investigated new voltage regulation strategies for distribution system operators (DSOs) to lessen the DG and load variation impact on grids [18,19,20,21]. The voltage regulation schemes can be classified into different categories according to the communication architecture [21]: local control, in which no coordination is deployed but only measurements at the point of common coupling are used; centralized control, in which the measurements from the grid are gathered to calculate the optimal setpoints that are sent to the distributed systems; distributed control, in which a global system does not control the distributed intelligent systems, but they interact only with the neighboring devices to reach pre-determined goals; decentralized control, which consists of a hybrid solution between centralized and distributed control. 

The different voltage regulation schemes are deeply analyzed in the literature, but the cybersecurity aspects are often neglected. For example, in Ref. [22], a decentralized control system is investigated by considering the dynamic voltage regulation of a hybrid distribution transformer. In [23], DG systems’ capabilities are exploited to keep voltages within specified limits in the context of a combined local and centralized voltage control. The control architecture is demonstrated on a 75-bus test system hosting 22 DG units. In refs. [24,25,26,27], centralized voltage control schemes are adopted to maintain voltage stability in an active distribution grid context. The voltage regulation levels are voltage source converters (VSCs), considered reactive power sources, on-load tap changers (OLTC), and step voltage regulators (SVRs). For example, in [24], the voltage fluctuations forecast is used to optimize the tap position, maximizing the minimum voltage margin from the voltage limits.

In ref. [28], the centralized voltage control is analyzed considering the data falsification attacks’ impact. In detail, the attacks are related to the voltage measurements transmitted from the field to the control system. The authors built up a two-step method based on machine learning techniques to detect false data injection in the context of an active grid. The proposed method was tested on a 240-bus real distribution system and the standard IEEE 123-node benchmark distribution network.

In ref. [29], a volt–var control system object of a cyberattack is investigated considering different knowledge levels of the attacker about the control system, network topology, and monitoring system. The authors formulated a game between the malicious attacker and the operator, from which possible countermeasures can be derived to detect and mitigate the attack.

In ref. [10], the impact of false data injection on a centralized voltage control system is studied in a distribution grid characterized by a high level of PV penetration. The authors demonstrated that the falsified measurements could provoke a relevant number of voltage violations. The detection algorithm suggested in [10] is analyzed in a residential area with one feeder.

The impact of cyberattacks on the remedial action schemes of large transmission systems is investigated in [30], proposing different metrics for evaluating the effects of malicious operations. The simulations are made on synthetic Illinois 200-bus and South Carolina 500-bus systems by means of a zonal equivalent model. 

In ref. [31], a formulation for detecting and characterizing cyberattacks is proposed in the context of a control center of a transmission system network. The authors investigate the impact of false data related to the parameters involved in calculating the optimal power flow necessary for grid management.

Different from the other surveyed papers, this paper aims to evaluate the FDIA impact by developing a false data generator that should simulate capillary penetration of the attacks and their effects in the context of a large DN. This paper presents a voltage regulation framework based on the microgenetic algorithm (μGA), which centrally controls the voltage sending optimized setpoints to DG units to maintain the voltage levels within limits; moreover, it investigates the impact of false data injection in the context of complex distribution systems in which the voltage regulation effectively manages network voltage. The IEEE 118-bus test system is tested in MATLAB, considering different types of false data attacks. These attacks are generated using a false data generator developed for this research; unlike other surveyed papers, the false data are not manually defined and more than one typology is evaluated.

The main contributions of this paper are the μGA-based voltage regulation framework; a false data generator—suggested to provide reasonable false datasets related to power profiles of residential and non-residential customers; and the analysis of the impact of false data injection in the context of a complex distribution grid reporting a relevant number of different scenarios.

The paper is organized as follows: Section 2 describes the developed false data generator model. Section 3 reports the developed centralized voltage regulation framework to be tested against a case study presented in Section 4. Section 5 presents the simulations’ results considering different combinations of anomalies and the percentage of manipulated load profiles. Section 5 concludes the paper.

## 2. False Data Generator Model

The false data generator was realized through a series of functions in a Python environment. Starting from a dataset, a portion of the data is altered with different typologies of anomalies, and a false dataset is returned. The developed generator is flexible and does not require further adaptions if different profiles are considered.

The model is based on the data frames of the Pandas module, in which each variable in the data frame corresponds to a different electrical bus in which the values are to be analyzed. 

Once it has been identified which users are experiencing data falsification, anomalies are created by acting on a fraction of points in the dataset relating to an electrical user (which may be a generation plant or load) and modifying their values according to random parameters from preset lists.

The false data generator requires various parameters before creating the anomaly:

- The type of anomaly: spot, drift, or mixed.
- The percentage of users subject to the cyberattack, N_false%_.
- The fraction of anomalies present per node, N_false%_.
- Anomaly scale parameter, σ(N_K_).
- The fraction of anomalies parameter, φ(N_K_).
- Mean drift parameter, μ(N_K_).
- Drift spread parameter, γ(N_K_).

**Figure 1:**
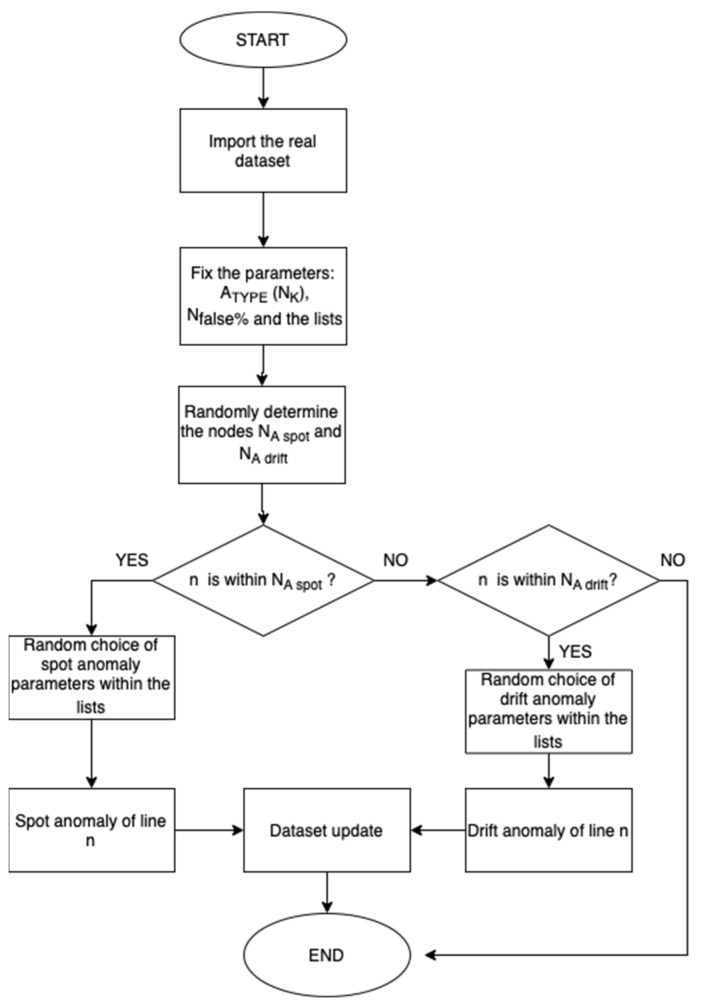
False data generator algorithm.

The process of creating the anomaly is shown in Figure 1.

For **spot-type** anomalies, first, the algorithm evaluates the minimum and maximum values of the input array related to a specific node N_K_, from which the amplitude values can be obtained as:

(1) $${∆}_{arr}(N_{K})={Arr}_{max}(N_{K})-{Arr}_{min}(N_{K})$$

Based on the φ(N_K_) parameters obtained by randomly extracting from the list, φ_list_, containing the permitted values for the anomaly fraction, a portion of the timestamps is modified, extracted randomly by an amount equal to:

(2) $$N_{timestamps}\left(N_{K}\right)=\varphi (N_{K})\times t$$

These data are randomly increased or decreased according to the following formula:

(3) $${x(N_{K})}_{t}^{False}={x(N_{K})}_{t}^{Real}\pm \sigma (N_{K})\times {}_{arr}(N_{K})$$

**Drift-type** anomalies vary the magnitude by raising or lowering the arrays’ mean value from a specific time. First, a drift time, τ_drift_, is set, chosen randomly within the analysis period Δt, at which the value of the quantity undergoes a shift, then the values of μ(N_K_) and γ(N_K_) are defined, both obtained by randomly extracting from the lists γ_list_ and μ_list_, and the first represents the percentage of mean drift, while the second represents the percentage change in the spread of the data. 

In this type of anomaly, for the values before τ_drift_, the magnitude remains the same, while for the values after τ_drift_, the new value can be evaluated as:

(4) $${x(N_{K})}_{t}^{False}={x(N_{K})}_{t}^{Real}+\frac{\sum_{\tau_{drift}}^{N_{after}}{x(N_{K})}_{t}^{Real}}{\left(N_{after}-\tau_{drift}\right)}\times \frac{\mu \left(N_{K}\right)}{100}\times \left(1+\frac{\gamma \left(N_{K}\right)}{100}\right),$$

where N_after_ represents the number of timestamps after the drift time.

**Figure 2:**
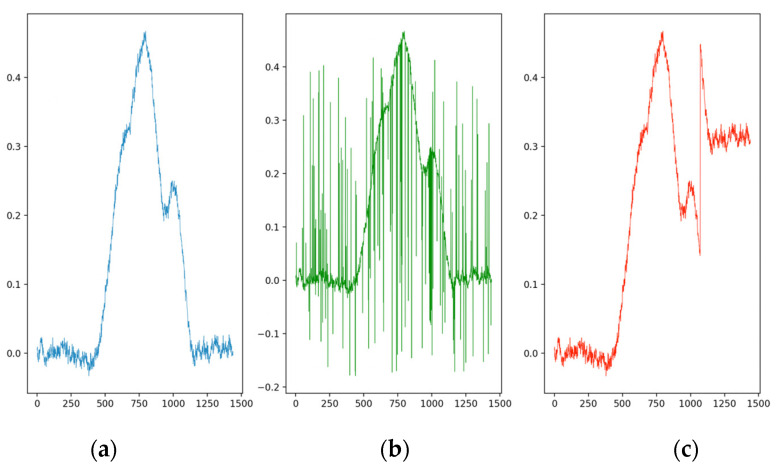
(**a**) Real trend; (**b**) trend with spot anomalies; and (**c**) trend with drift anomalies.

In the case of a **mixed typology** anomaly, 50% of the nodes involved have a spot data falsification, while the remaining 50% have a drift-type falsification. In Figure 2, two typologies of anomalies are shown.

## 3. Centralized Voltage Regulation Framework 

The centralized voltage regulation system model is based on the possibility of remotely changing the operating points of DG plants to sustain the grid’s voltage profile. Due to the increasing penetration of DG, it is expected that operators could adopt such a voltage regulation framework to avoid many interruptions caused by exceeding the voltage limits. In detail, in the future, the reactive power exchange could be adjusted by the operator without affecting the active power production, according to the limits fixed by the capability diagram of the renewable-based generators, as it is detailed in [32]. In the context of demand-side management, it is expected that some flexibility for network services could be exploited in an automatic way. 

With respect to regulation support from DGs, similar mechanisms are in progress in some existing networks, or they are defined in standards; for instance, the Italian grid codes define a set of DG that can be automatically disconnected by the operators in order to ensure the system security [33]. According to the German grid code for connection to the LV grid, new PVs with less than 30 kW capacity that cannot be controlled remotely have to limit their output to 70% of rated power; the remote control of active power output at the request of the system operator is required for all DER rated above 100 kW connected to the grid [34]. According to the Chinese energy storage connection code GB36547-2018, the system operator can send setpoints and should work for suitable voltage management. The current version of IEEE Std 1547 [34] specifies a requirement for all conformant DER to respond to (local and/or remote) control signals limiting the active power; furthermore, there is no DER size threshold for this requirement.

In this paper, it was assumed that the regulation framework could act on a medium voltage network; generators are connected to the SSs of the MV network under analysis. 

The regulation framework is implemented in the Octave environment using the MATPOWER package, which calculates load flows according to the network topology and active power (P) and reactive power (Q) values assigned to each node. Indeed, an SS is modeled as a P and Q node in the load flow analysis, considering the local power consumption and production contributions.

**Figure 3:**
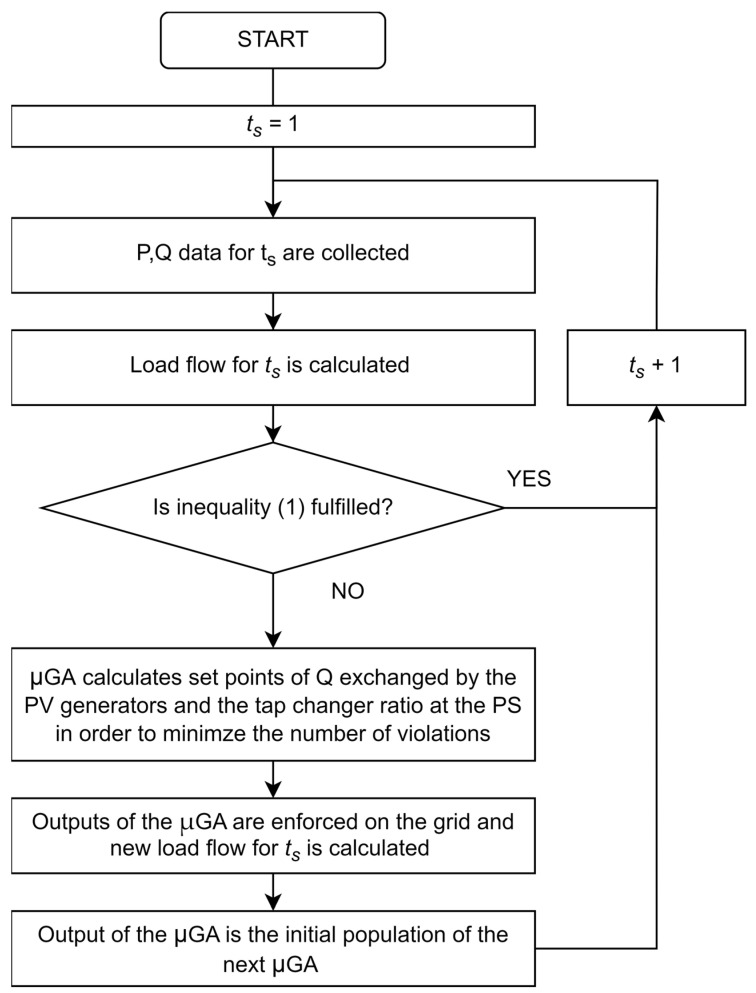
Flowchart of the voltage regulation framework.

The main steps of the regulation framework are presented in Figure 3. Firstly, load flows are calculated for the analyzed timestamp; then, the voltage regulation is activated if the following inequalities are not fulfilled for each node of the network:

0.95 ≤ V_i_ ≤ 1.05, ∀i ∈ [1, N_SS_] (5)

where V_i_ is the voltage at the SS characterized by the index i.

According to Figure 3, if inequality (5) is not fulfilled, the regulation is activated. The implemented regulation exploits the reactive production or absorption of renewable-based generators. The reactive power exchanges have to fulfill the typical capability diagram of the PV generator, as in [35]. In detail, the maximum Q that a DG system can provide (Q_max_) to the grid should be calculated according to the following equation, considering an operating point characterized by an active power production greater or equal to 10% of the apparent power S_n_:

Q_max_ = P_n_ ∙ 0.484 (overexcited mode) (6)

where P_n_ is the nominal power of the DG system.

The minimum Q absorbed by the DG system (Q_min_) is calculated with the following equation:

Q_min_ = −P_n_ ∙ 0.484 (under excited mode) (7)

**Figure 4:**
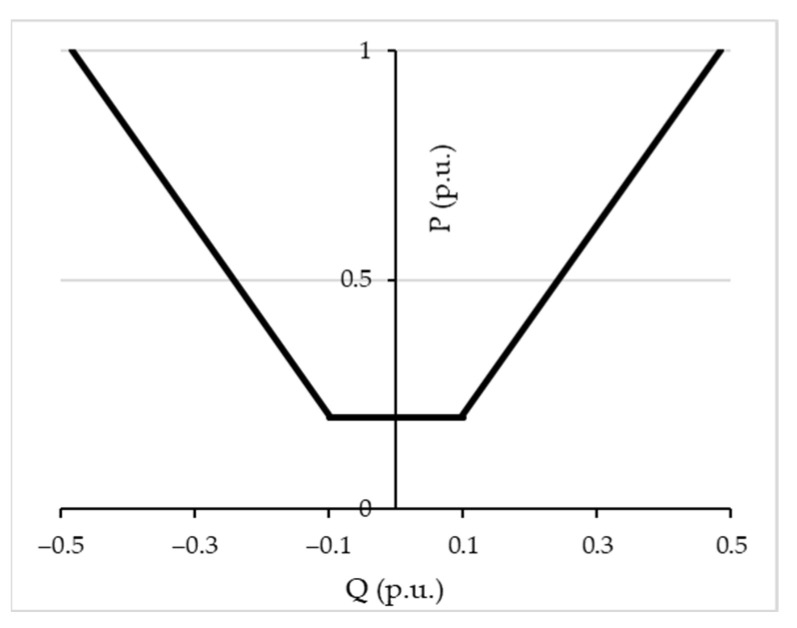
Capability curve assumed for DG in the voltage regulation framework.

When the active power production is under 10% of the P_n_, the reactive power cannot be exchanged with the grid. These limits are also shown in Figure 4.

The voltage regulation model acquires the active and reactive profiles of load and generation by each SS and performs the load flow, considering the topology of the grid and the interconnection with the HV system as the reference bus. Then, according to inequality (5), the total number of violations is calculated. In case of violation occurrence, the model tries to reduce the number of violations using the reactive power support by DG. 

As reported in Figure 3, the optimization technique used for this nonlinear problem is based on a microgenetic algorithm, implemented by the authors in the open-source Octave environment and coupled with MATPOWER. The µGA was developed by the authors to solve other research issues, as in [35]. The usage of µGA enables addressing the solution of nonlinear problems with adequate performance, even during real-time operation.

The algorithm initializes a random sample of individuals with the values in p.u. of reactive power for each SS to be optimized. The evolution via survival of the fittest is adopted, and the selection scheme used is tournament selection with a shuffling technique for choosing random pairs for mating. The routine includes binary coding for individuals, jump mutation, creep mutation, and the option for a single-point crossover; a restart mechanism with elitism is also implemented. The population size is fixed to five individuals. 

Each individual has a number of genes equal to the N_SS_ + 1; N_SS_ describes the reactive power exchanges of the generators, while the last gene corresponds to the tap changer of the transformer of the PS.

The objective function to be minimized by the μGA is defined as the number of violations. Two stopping criteria are implemented: the algorithm is stopped as soon as the number of voltage violations is nihil or the maximum number of generations is reached. Therefore, in each iteration, the μGA performs the load flow of the DN to evaluate the number of remaining violations after implementing the combination of setpoints in terms of reactive power exchanged by DG systems. It is worth highlighting that the performances (i.e., a reduced number of generations and the related execution times) are dramatically improved by exploiting the solution of the previous iteration for the current calculation. Leveraging this recursive behavior, μGA suggests a minimum amount of changes; moreover, generations start only if the solution of the previous timestamp causes a violation during the new timestamp. The entire procedure is repeated for each timestamp according to the case study.

In order to evaluate the voltage deviations, the following indices are calculated after the execution of the VR:

- TV_1.05_ is the number of timestamps during which the maximum network voltage is higher than 1.05.
- TV_1.1_ is the number of timestamps during which the maximum network voltage is higher than 1.1.
- TV_1.15_ is the number of timestamps during which the maximum network voltage is higher than 1.15.

## 4. Case Study

**Figure 5:**
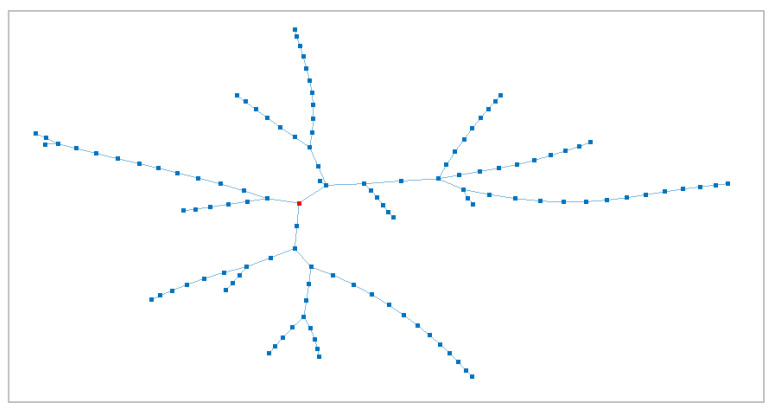
Case study topology (red marker corresponds to the PS).

The impact of false data on voltage regulation is tested in a case study, namely, a 118-node distribution network, as described in [36]. Its topology is reported in Figure 5; the figure first highlights the radial structure with four main feeders and a PS, identified by a red square marker; each node corresponds to a SS with some connected customers. This case study was identified as an interesting test system due to the presence of long feeders. This a typical configuration in which voltage issues could arise in the case of large reverse power flows. This case study is not accompanied by current limits on the branches; therefore, when higher flowing currents are considered (i.e., assuming a certain penetration of DG), it was assumed that these are within the line limits and protection thresholds.

After the definition of the topology, power profiles were defined. Concerning the passive loads, a yearly load profile was considered according to [37]. This is a profile in p.u.; therefore, it was scaled up according to the loads assigned to the buses in the original network in [36]. Moreover, a coefficient that randomly varies from 0.85 to 1.15 was applied. Applying these profiles, it was assumed that the loads absorb 290 MWh yearly while the average peak load of nodes is 0.19 MW.

In addition to the existing passive loads, PV generators were added to all nodes. Concerning the power profiles of these generators, an open-access dataset was exploited [38]. The plant sizes are crucial parameters for defining the impact of distributed generators on voltage profiles so that a regulation would be frequently enforced. In this respect, a massive deployment of PV plants that can supply about 40 % of the energy consumption (i.e., the rest of the energy is provided by the transmission grid using the PS) was evaluated, assigning a size from three to four times the peak power of the load profile of each node. The installed generators produce 444 MWh yearly, supplying 120 MWh to the loads, and 324 MWh would be the resulting reverse power flow at the PS. Although the simulated conditions are dramatic, they represent those areas with low load concentration but high potential for RES installation (e.g., good exposure, a lack of authorization constraints, free lands, and massive penetration of agrophotovoltaic plants) in combination with a weak network that could suffer voltage arising. Indeed, their network operators are already reporting a continuous reverse flow at their PSs.

**Figure 6:**
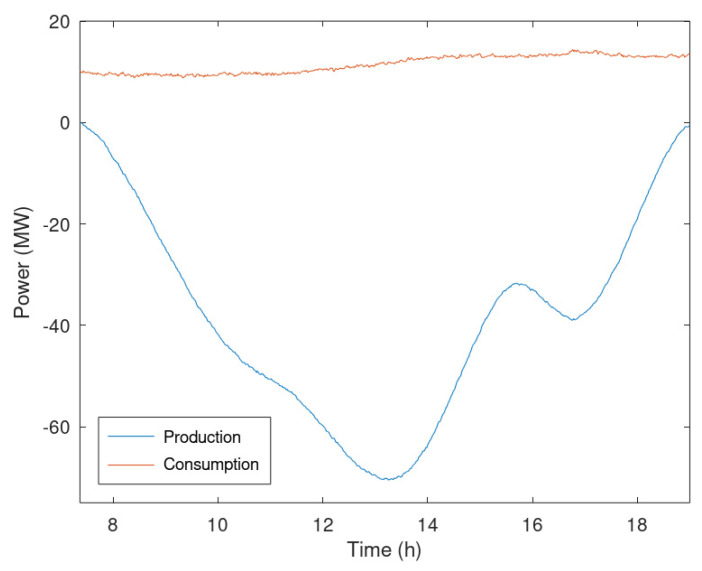
Global power profiles in case of production peak used in the case study.

After defining the reference profiles and the sizes to be considered, production and consumption profiles were sampled every minute. For clarity, the analysis focuses only on one day during which the reverse power flow was maximum, and maximum voltage deviations were detected. Therefore, the global consumption and production under analysis are provided in Figure 6.

Results were gathered by simulating the VR carried out every minute based on the power profiles of the previously mentioned SSs. Indeed, the effect on voltage profiles was first calculated when the received data were correct; secondly, various sets of false data were applied to evaluate their negative effect on the regulation efficiency and the network itself.

**Figure 7:**
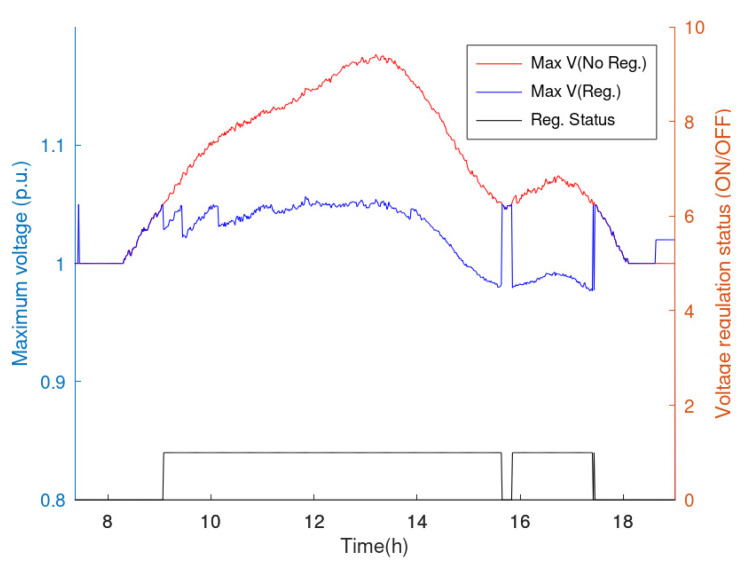
Case study voltage regulation.

According to the previous section, the VR framework receives as input P and Q of the nodes and the active power injected by the generators; these profiles are falsified to test the effects of FDIA. Moreover, voltage regulation can leverage the transformer ratio at the PS (i.e., varying the secondary voltage at the PS) and the reactive power exchanged by PV generators. Assuming that the secondary voltage at the PS is equal to 1 p.u., voltage regulation is active as soon as the voltage is higher than 1.05 p.u. or lower than 0.95 p.u. Figure 7 reports the trends of maximum and minimum voltage if regulation is applied. When regulation is not active, voltages are assumed to be equal to those calculated for the basis scenario (i.e., regulation is not enforced and secondary voltage at the PS is 1 p.u.). It is worth highlighting that the maximum voltage is always lower than 1.1 p.u. because of regulation enforcement; indeed, the operators typically enforce this limit to detect some network violations. The beneficial effects of the VR framework can also be found by analyzing TV_1.05_, which corresponds to 67.8%, while when VR effects are calculated, this value decreases to 7.4%.

The attack scenarios simulated in this paper are characterized by distributed manipulation of consumption and production data at the SSs. In detail, the P and Q profiles of the production power plant and the overall power demand at SSs are manipulated. The results were calculated considering the two types of threats previously described (i.e., drift and spot anomalies) and their mixed combination. Moreover, an increasing amount of manipulated measurements was considered for the simulation; notably, it was simulated that 25, 50, or 75% of the nodes are affected by the anomalies. 

## 5. Results

This section presents the simulation results considering the case study and load profiles previously presented, assuming that an increasing number of profiles are manipulated. In this section, an evaluation of the impact of false data is carried out considering two main sets of simulations: the first set regards some attacks that manipulate up to 25% of the measurements, while the second set regards those attacks that massively manipulate the measurements; namely, 50 and 75% of the data are falsified. The results are presented by calculating mean values, the standard deviation of the voltages (SD), and the defined indices TV_1.05_, TV_1.05_, and TV_1.05_.

### 5.1. FDIA Affecting 25% of the Measurements

The first set of simulations regards the effects of manipulating 25% of the measurements applying a mixed anomaly; 10 simulations randomly assigned the set of profiles to be manipulated. The overall results are shown in Table 1, which reports the average values collected from the 10 simulations; furthermore, these are compared with the values calculated when the voltage regulation framework does not exploit manipulated data. Considering the statistical parameters, it can be highlighted that the manipulated data cause notable effects on voltage regulation; on average, 6% of the timestamps report voltage violations on the network (i.e., voltage overcomes 1.1 p.u. in at least one node). It is worth highlighting that false data are processed during all the timestamps; therefore, the VR framework can still solve voltage issues. Indeed, considering that the lack of VR leads to TV_1.05_ higher than 1.05, equal to 67.8%, on average, 30% of the voltage violations are still solved by the regulation procedure.

**Figure 8:**
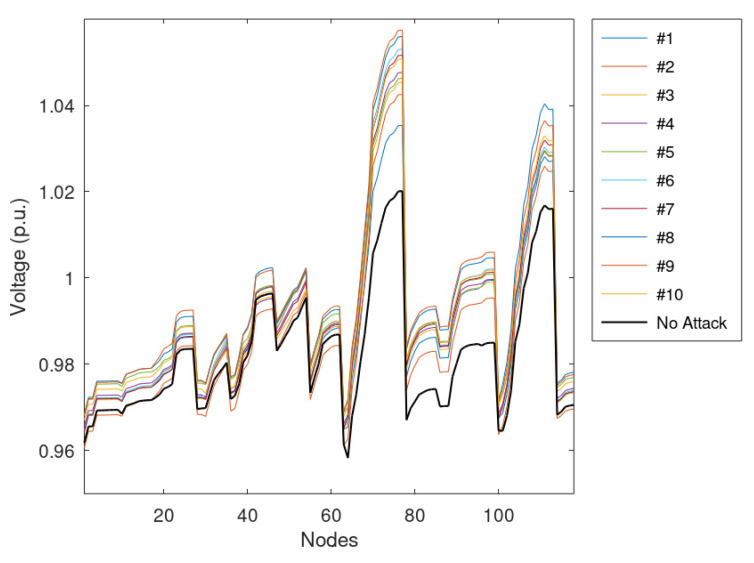
Average voltage of the nodes assuming that 25% of measurements are manipulated.

The average voltage of the nodes is reported in Figure 8. The figure shows the individual effects of the 10 simulated anomalies and the profile calculated without manipulation, which is taken as a reference. Figure 8 highlights that the longest feeders (i.e., those with the highest numbers of nodes) have the highest voltages and the highest differences among results (i.e., the maximum difference is about 0.03 p.u.). Moreover, some nodes have a voltage lower than the reference; this behavior is due to the lack of disruptive anomalies on the feeder that do not jeopardize the VR framework. 

**Figure 9:**
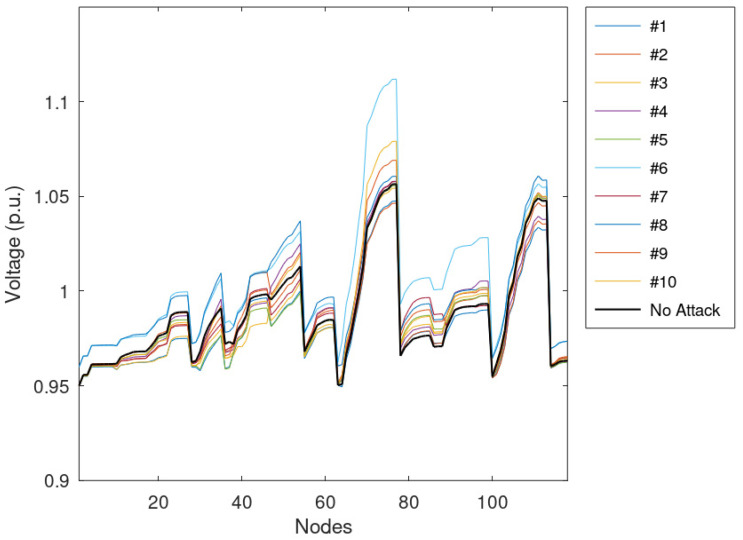
The voltage of the nodes during the maximum violation, assuming that 25% of measurements are manipulated.

A specific timestamp is also reported in Figure 9 as an example that shows the voltages calculated during the timestamp associated with the maximum voltage in the case of real data. In this case, a violation on voltage higher than 1.1 p.u can be seen.

**Figure 10:**
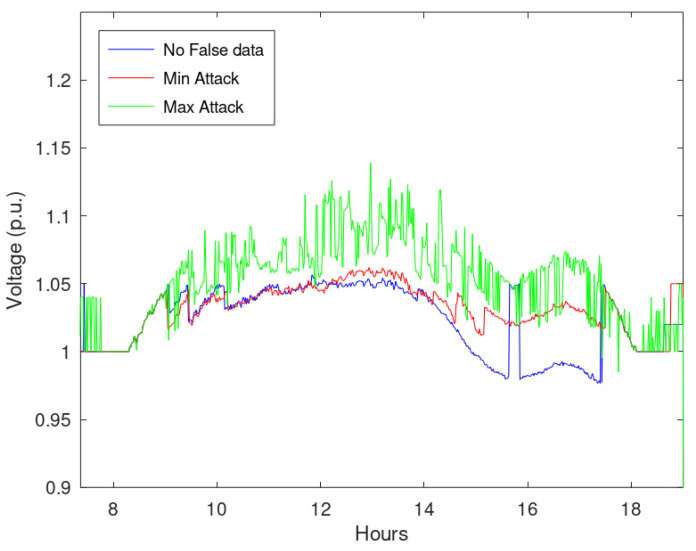
Maximum network voltage during the timestamps considering maximum and minimum violations, assuming that 25% of measurements are manipulated.

In addition, Figure 10 shows the maximum network voltage during the simulated time period, considering the false data that cause maximum and minimum violations, identified as Min and Max Attack, respectively. This figure highlights that a specific combination of false data does not cause voltage violations, while the most disruptive falsification leads to violations.

### 5.2. Massive Spread of FDIA

An additional set of simulations regards the effects of an increasing amount of falsified measurements. Notably, it simulated the effects of spot and drift anomalies when applied to 50 or 75% of measurements. The main results of these simulations are collected in Table 2, which shows the notable impact on the voltages caused by incorrectly processed data; in particular, calculated violations overcome even 1.15 p.u.

**Figure 11:**
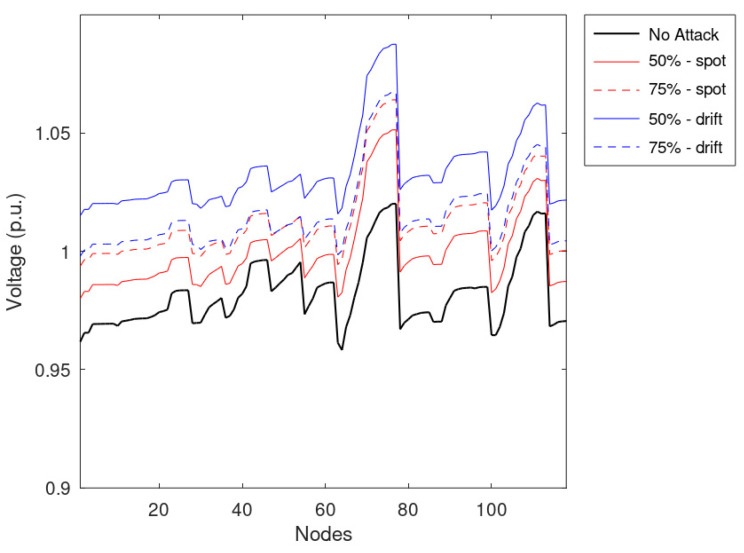
Average voltage of the nodes, assuming that 50 and 75% of measurements are manipulated.

**Figure 12:**
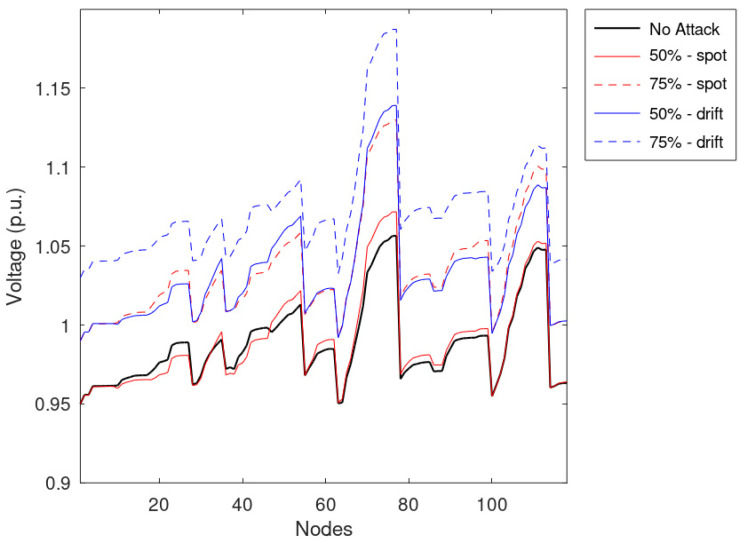
The voltage of the nodes during the maximum violation, assuming that 50 and 75% of measurements are manipulated.

Similarly to the previous simulations, the average voltages of the nodes are reported in Figure 11, while Figure 12 shows the voltages during the most critical timestamps identified during the regular operation. Considering Figure 12, it can be noted that the spot anomaly does not cause any voltage issues on the network if applied to half of the measurements; the drift anomaly has a higher impact on the network, causing overvoltages. Moreover, considering the average effects on the network presented in Figure 10, it can be shown that drift anomalies can have a higher impact on the distribution networks jeopardizing the VR framework. It is worth noting that the profile associated with a lower number of falsified profiles produced a higher voltage profile on average, even if violations are more frequent in the case of a higher number of falsified profiles. Indeed, a more comprehensive set of falsified measurements leads to higher standard deviation values; namely, overvoltages are more frequent and more intense. Moreover, by taking advantage of the evaluation of a wide set of values, it was found that the distribution of false profiles that introduce low measurement variations is more dangerous than concentrated threats that introduce the highest differences from the actual measurement.

**Figure 13:**
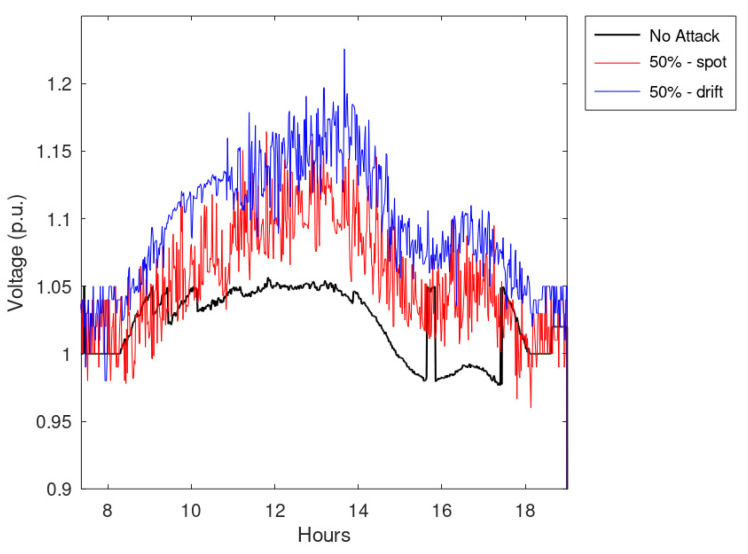
Maximum network voltage during the timestamps considering half of the measurements falsified.

**Figure 14:**
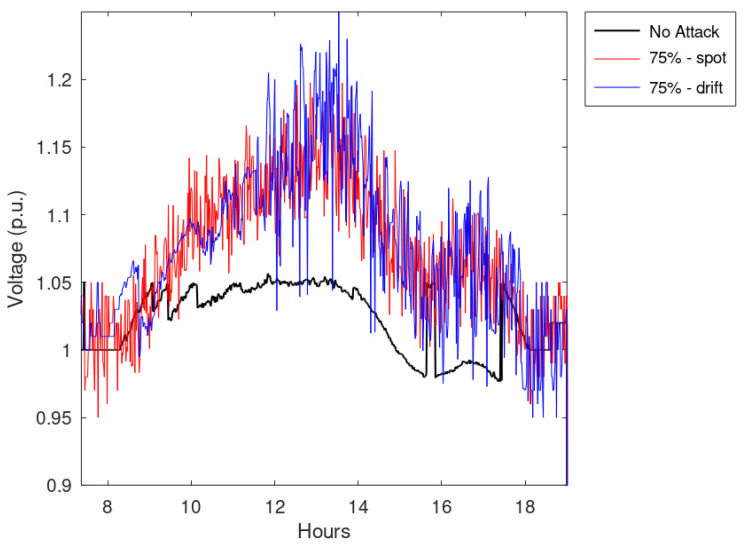
Maximum network voltage during the timestamps considering 75% of the measurements falsified.

Finally, Figure 13 and Figure 14 report the maximum network voltages calculated for all the timestamps. These figures show that the effects of drift anomalies are more disruptive than the spot anomalies. 

## 6. Conclusions

This paper evaluated the effects of a false data injection attack on a voltage regulation framework. A GA-based VR framework was developed and implemented on a test network to assess these effects, for which a broad penetration of PV plants was simulated to test the beneficial effects of the regulatory framework. Data manipulation was provided by developing a false data generator based on spot and drift anomalies and their combination.

The main results were gathered by simulating an increasing amount of falsified data and applying the random falsification introduced by the false data generator, assuming that the detection system was unable to detect it or that a detection system was not used. According to the simulation results, it can be stated that an increasing amount of falsified data led to undesired overvoltages that are caused by wrong signals sent to PV generators, which can modify their exchanged reactive power. When a reduced amount of manipulated data was considered, it can be noted that some manipulation combinations did not lead to overvoltages: the attack reduced system efficiency and operator awareness without jeopardizing the service. In particular, the VR framework showed promising performances since some voltages arising were avoided, even when wrong measurements were processed. It was found that the distribution of false profiles that introduce low measurement variations was more dangerous than concentrated threats that introduce the highest differences from the actual measurement. Moreover, during the simulations, it was found that drift anomalies could be more disruptive than spot anomalies. Therefore, the new smart distribution networks should be resilient by design against false data injection attacks, considering their dramatic effects on system reliability.

In future works, the authors would like to compare the results when different voltage regulation mechanisms are implemented, assessing their resilience in combination with a false data detection algorithm.

## Figures and Tables

**Table 1:** Main results comparing average effects of 10 simulations with 25% of manipulated measurements and true data processed by VR framework.

	Max	Min	Mean	SD	TV_1.05_	TV_1.1_
**True Data**	1.056	0.941	0.982	0.0233	7.4%	0.0%
**Attack on 25% Nodes**	1.127	0.945	0.991	0.0261	31.5%	6.0%

**Table 2:** Main results considering a combination of anomalies and percentage of falsified measurements.

	True Data	Attack on 50% of Measurements		Attack on 75% of Measurements	
Manipulation Type	N/A	Spot	Drift	Spot	Drift
**Max**	1.056	1.164	1.181	1.252	1.215
**Min**	0.941	0.923	0.941	0.886	0.891
**SD**	0.023	0.029	0.028	0.040	0.039
TV_1.05_	7.4%	48.4%	64.4%	63.7%	65.1%
TV_1.1_	0.0%	15.0%	29.6%	31.4%	31.0%
TV_1.15_	0.0%	0.6%	2.3%	11.7%	13.3%

## Data Availability

This article exploits existing open datasets.

## Abbreviations

γ(N_K_)	Drift spread parameter, used for drift anomaly. It represents the percentage change in the spread of the data for the node N_K_.
γ_list_	List of all possible values for the spread drift parameter.
Δ_arr_(N_K_)	Maximum variation in values for the array relative to the N_K_ node
Δt	Analysis time-period.
φ(N_K_)	Fraction of anomalies parameters for the node N_K_, used for the spot anomaly. It represents the number of node values subject to the spot anomaly.
φ_list_	List of all possible values for the anomaly fraction parameter.
μ(N_K_)	Mean drift parameter used for drift anomalies. It represents the percentage of mean drift for the node N_K_ after the drift anomaly starts.
μ_list_	List of all possible values for the anomaly mean drift parameter.
σ(N_K_)	Anomaly scale parameter for the node N_K_, used for the spot anomaly. This factor will scale the range of the normal data to inject anomalies.
σ_list_	List of all possible values for the anomaly scale parameter.
τ_drift_	Used for drift anomalies, it represents the time at which drift starts.
A_TYPE_ (N_K_)	Typology of anomaly attack at the node N_K_.
Arr_min_(N_K_)	Minimum value of the array referring to node N_K_ data.
Arr_max_(N_K_)	Maximum value of the array referring to node N_K_ data.
N_after_	Number of timestamps after the drift time for the drift anomaly.
N_A spot_	Number of nodes subject to spot anomalies.
N_A drift_	Number of lines subject to drift anomalies.
N_false%_	Percentage of lines subject to data falsification.
N_false_i_	Indexes of nodes subject to data falsification.
N_timestamp_(N_K_)	Number of timestamps modified by the spot anomalies.
V_i_	Voltage of the i-th node.
${x(N_{K})}_{t}^{Real}$	Real value of the array related to the node N_K_ in the timestamp t.
${x(N_{K})}_{t}^{False}$	False value of the array related to the node N_K_ in the timestamp t.
